# Quality of life, socioeconomic and clinical factors, and physical exercise in persons living with HIV/AIDS

**DOI:** 10.1590/S1518-8787.2017051006266

**Published:** 2017-07-07

**Authors:** Rafaela Catherine da Silva Cunha de Medeiros, Jason Azevedo de Medeiros, Tatiane Andreza Lima da Silva, Ricardo Dias de Andrade, Danielle Coutinho de Medeiros, Juliany de Souza Araújo, Antônio Manuel Gouveia de Oliveira, Marcos Aurélio de Albuquerque Costa, Paulo Moreira Silva Dantas

**Affiliations:** I Programa de Pós-Graduação em Ciências da Saúde. Universidade Federal do Rio Grande do Norte. Natal, RN, Brasil; II Departamento de Educação Física. Universidade Federal do Rio Grande do Norte. Natal, RN, Brasil; III Curso de Educação Física. Centro Universitário do Rio Grande do Norte. Natal, RN, Brasil; IV Curso de Educação Física. Centro Universitário FACEX. Natal, RN, Brasil; V Programa de Pós-Graduação em Educação Física. Universidade Federal do Rio Grande do Norte. Natal, RN, Brasil; VI Departamento de Farmácia. Universidade Federal do Rio Grande do Norte. Natal, RN, Brasil

**Keywords:** HIV Long-Term Survivors, Socioeconomic Factors, Health Behavior, Quality of Life

## Abstract

**OBJECTIVE:**

To analyze whether socioeconomic and clinical aspects and the aspects of healthy life habits are associated with the quality of life of persons living with HIV/AIDS.

**METHODS:**

This is a cross-sectional exploratory quantitative research, with 227 persons living with HIV/AIDS, treated at two hospitals of reference between April 2012 and June 2014. We used structured questionnaires to assess socioeconomic aspects (gender, age, education level, marital status, race, socioeconomic status, dependents on family income, employment relationship), clinical parameters (time of disease diagnosis, use and time of medication, CD4 T-cell count, and viral load), and practice of physical exercise. To assess quality of life, we used the Quality of Life questionnaire (HAT-QoL). For characterization of the socioeconomic and clinical data and domains of quality of life, we conducted a descriptive analysis (simple frequency, averages, and standard deviations). We applied linear regression, following a hierarchical model for each domain of quality of life.

**RESULTS:**

The domains that presented lower averages for quality of life were financial concern, concern with confidentiality, general function, and satisfaction with life. We found associations with the variables of socioeconomic status and physical exercise, therapy, and physical exercise for the last two domains, consecutively.

**CONCLUSIONS:**

The quality of life of persons living with HIV/AIDS shows losses, especially in the financial and confidentiality areas, followed by general function of the body and satisfaction with life, in which socioeconomic and clinical aspects and healthy living habits, such as the practice of physical exercise, are determining factors for this reality.

## RESUMO

**OBJETIVO:**

Analisar se aspectos socioeconômicos, clínicos e de hábitos de vida saudável estão associados à qualidade de vida em pessoas vivendo com HIV/aids.

**MÉTODOS:**

Pesquisa quantitativa exploratória de corte transversal, com 227 pessoas vivendo com HIV/aids, atendidos em dois hospitais de referência entre os períodos de abril 2012 a junho de 2014. Foram utilizados questionários estruturados para avaliar aspectos socioeconômicos (sexo, idade, escolaridade, estado civil, cor de pele, *status* socioeconômico, dependentes da renda familiar, vínculo empregatício), parâmetros clínicos (tempo de diagnóstico da doença, uso e tempo de medicação, contagem de células TCD4 e carga viral) e prática de exercício físico. Para avaliar qualidade de vida, utilizou-se o questionário *Quality of Life* (HAT-QoL). Para caracterização dos dados socioeconômicos, clínicos e domínios da qualidade de vida, conduzimos análise descritiva (frequência simples, médias e desvios-padrão). Aplicamos regressão linear, seguindo um modelo hierárquico para cada domínio da qualidade de vida.

**RESULTADOS:**

Os domínios que apresentaram menores médias para a qualidade de vida foram preocupação financeira, preocupação com sigilo, função geral e satisfação com a vida. Foram encontradas associações com as variáveis *status* socioeconômico e exercício físico; terapia; e exercício físico para os dois últimos domínios, consecutivamente.

**CONCLUSÕES:**

A qualidade de vida de pessoas vivendo com HIV/aids apresentam prejuízos, principalmente nas questões financeiras e de sigilo, seguidos da função geral do corpo e satisfações com a vida, em que os aspectos socioeconômicos, clínicos e hábitos de vida saudável, como a prática de exercício físico, são fatores determinantes para essa realidade.

## INTRODUCTION

Acquired Immunodeficiency Syndrome (AIDS), initially considered an acute and fatal disease, currently has a profile of chronic disease^1^. This modification is due to the highly active antiretroviral therapy (HAART), which has allowed an increase in the quality of the treatment of persons living with HIV/AIDS (PLWHA), improving the quality of life (QOL) and increasing life expectancy^2^.

The QOL covers multifactorial characteristics, reflecting individual and collective experience, knowledge, and values in the historical, cultural and social time lived^3^. In its comprehensive concept, QOL is defined as an individual perception about the position in life, in the context of culture and value systems in relation to goals, expectations, and concerns^4^. In this perspective, we can verify the incorporation of aspects related to the physical and psychological health, the level of independence, and the social relationship within the spectrum of QOL^4^.

Given these characteristics, the investigation on the QOL of PLWHA becomes essential, especially because of the development of the infection, the need for drug treatment, increased survival, and life with the stigmatizing disease. All these aspects must be observed with the socio-economic characteristics and habits of life, in order to identify negative factors that contribute to biopsychosocial problems^3^ ^,^ ^5^ ^,^ ^6^.

Given this context, this study aimed to analyze whether socioeconomic and clinical aspects and the aspects of healthy life habits are associated with the quality of life of persons living with HIV/AIDS.

## METHODS

This is an exploratory descriptive cross-sectional study^7^, with persons living with HIV/AIDS treated at two hospital of reference in infectious diseases of the State of Rio Grande do Norte, Brazil: Hospital Giselda Trigueiro, in the municipality of Natal, and Hospital Rafael Fernandes, in the municipality of Mossoró, carried out between April 2012 and June 2014. The study was approved by the research ethics committee of the Universidade do Estado do Rio Grande do Norte (Opinion 648,909).

The sample was non-probabilistic and consecutive; among the 275 patients approached and invited to participate in the research by the evaluators after medical consultation, 46 refused because of unavailability of time or lack of interest in participating, and two did not complete the interview. Thus, 227 persons living with HIV of both genders participated in this study.

Inclusion criteria were: diagnosis of infection of HIV/AIDS, being in clinical follow-up in the Specialized Care Service in HIV/AIDS, and being aged 18 years or more, of both genders. We excluded individuals who had any type of medical or cognitive condition that would prevent them from understanding the research procedures (persons with severe hearing impairment, under the effect of psychoactive substance, or limitations in the ability to respond unaccompanied to the questionnaire, as in the case of prisoners with police escort or hospitalized).

The data were collected from personal interviews in a reserved location. The research tools were applied by two previously trained evaluators, using structured questionnaires, which assessed the socioeconomic and demographic aspects (gender, age, education, marital status, race, socioeconomic status, dependents on family income, employment relationship, and practice or not of physical exercise as a healthy habit).

Regarding clinical parameters, we inquired about the time of disease diagnosis and the use and time of medication. Data on clinical stage of infection, CD4 T-cell count, and viral load were obtained from the most recent exam record in the chart.

To assess the QOL, we used the questionnaire Quality of Life (HAT-QoL), proposed by Holmes and Shea^8^, validated for PLWHA in Brazil^9^. The HAT-QoL has 34 questions, distributed in nine domains: general function, satisfaction with life, health concern, financial concerns, concerns about medication, acceptance of HIV, concerns about confidentiality, confidence in the professional, and sexual function.

To answer the questions, subjects were told to think about their QOL in the last four weeks. The answers of the instrument have a five-point Likert scale: all the time, most of the time, part of the time, little time, never. In each domain, zero is the lowest score and 100 is the best possible score. Thus, the higher the score, the lower the impact of HIV infection is in the QOL of individuals.

### Statistical Analysis

We calculated the frequencies, averages, and standard deviations of the socioeconomic, clinical, and score data of the domains of quality of life. Subsequently, we selected the variables to be included in multiple regression models in order to eliminate the variables that showed no association with each domain of QOL from the model. In this analysis, Student’s t-test was used to compare the average scores for each domain between the levels of each variable, and the variables that had a value of p < 0.10 were selected for inclusion in the regression models.

For the multivariate analysis, multiple linear regressions were used. In each regression, the dependent variable was one of the domains of QOL and the independent variables were all the variables selected in the respective bivariate analysis. In each model developed, the independent variables that showed no significant association (p < 0.05) with the dependent variable were eliminated in a step-by-step process. The results are presented as adjusted point estimates of the difference in the score of each domain between the levels of each independent variable and their 95% confidence intervals.

## RESULTS

Of the 227 individuals, 131 were men and 96 were women, and average age of 40.3 (SD = 11.5) years. The characteristics of the population of the study are presented in Table 1, in which we can see that the most affected domains of QOL were financial concerns, concerns about confidentiality, general function, and satisfaction with life.

**Table 1:** Characterization of persons living with HIV/AIDS treated at the Specialized Care Service in HIV/AIDS. State of Rio Grande do Norte, Brazil, April 2012 to June 2014. (n = 227)

Characteristic	n	%
Education level (elementary and high school)	206	90.7
No common-law marriage	137	60.4
White	65	28.6
Socioeconomic status (class D-E - low)	144	63.4
Dependents on income		
1–3	142	62.6
4–10	85	37.4
No employment relationship	143	63.0
No practice of physical exercise	198	87.2
CD4 T-cell (average, standard deviation)	537.4	283.9
Undetectable viral load	150	66.1
Use of antiretroviral therapy	197	86.8
Diagnosis of HIV		
≤ 12 months of infection	46	20.3
> 12 months of infection	181	79.7
Time of medication		
≤ 12 months of medication	71	31.3
> 12 months of medication	156	68.7
Domains of quality of life (average, standard deviation)		
General function	59.3	26.2
Satisfaction with life	61.9	24.2
Health concerns	63.8	32.2
Financial concerns	38.9	35.9
Concerns about medication	73.8	24.1
Acceptance of HIV	66.3	37.9
Concerns about confidentiality	44.8	29.6
Confidence in the professional	86.6	20.0
Sexual function	76.2	35.2

The multiple linear regression model can be seen in Tables 2, 3, and 4. Associations were especially present between the variable “no practice of physical exercise” and the domains of general function, satisfaction with life, health concerns, acceptance of HIV, financial concerns, and concerns about medication. Among the socioeconomic variables, associations between age and the domains of health concerns, concerns about medication, and acceptance of HIV predominated.

**Table 2:** Multivariate analysis of the domains of quality of life of persons living with HIV/AIDS, treated at the SAE of Natal and Mossoró, State of Rio Grande do Norte, Brazil, from April 2012 to June 2014. (n = 227)

Variable	General function			Satisfaction with life			Health concerns		
		
	D	IC95%	p	D	IC95%	p	D	IC95%	p
Age	-6.24	-12.79–0.3	0.06	-	-	-	9.72	1.46–17.99	0.02
Education level	5.56	-1.18–12.31	0.1	6.37	-0.01–12.75	0.05	-	-	-
Physical exercise	-24.31	-34.06– -14.55	0.01	-14.99	-24.31– -5.67	0.01	-13.57	-25.93– -1.2	0.03
CD4 T-cell	5.55	-1.6–12.72	0.12	5.06	-1.72–11.85	0.14	9.93	0.87–18.98	0.03
Time of medication	-	-	-	-	-	-	3.48	-6.31–13.27	0.48

SAE: Specialized Care Service; D: difference

Significance level: p < 0.05.

**Table 3:** Multivariate analysis of the domains of quality of life of persons living with HIV/AIDS, treated at the SAE of Natal and Mossoró, State of Rio Grande do Norte, Brazil, from April 2012 to June 2014. (n = 227)

Variable	Acceptance of HIV			Financial concerns			Concerns about medication		
		
	D	IC95%	p	D	IC95%	p	D	IC95%	p
Gender	-	-	-	-8.51	-17.24–0.21	0.05	-8.51	-15.14– -1.88	0.01
Age	18.85	8.53–29.16	0.01	-	-	-	8.82	2.08–15.57	0.01
Education level	-	-	-	6.22	-3.18–15.63	0.19	-	-	-
Socioeconomic status	-	-	-	21.9	12.91–30.9	0.01	5.39	-1.42–12.2	0.12
Work	-	-	-	-2.57	-11.88–6.73	0.58	-	-	-
Physical exercise	-15.27	-29.65– -0.89	0.03	-27.05	-39.92– -14.2	0.01	-11.6	-21.4– -1.8	0.02
CD4 T-cell	-	-	-	-	-	-	7.37	0.09–14.65	0.04
Viral load	-	-	-	-	-	-	-2.45	-10.91–6.01	0.56
Time of medication	1.01	-10.04–12.07	0.85	-	-	-	-	-	-

SAE: Specialized Care Service; D: difference

Significance level: p < 0.05.

**Table 4:** Multivariate analysis of the domains of quality of life of persons living with HIV/AIDS, treated at the SAE of Natal and Mossoró, State of Rio Grande do Norte, Brazil, from April 2012 to June 2014. (n = 227)

Variable	Concerns about confidentiality			Confidence in the professional			Sexual function		
		
	D	IC95%	p	D	IC95%	p	D	IC95%	p
Gender	-	-	-	-	-	-	-20.94	-29.93–12	0.01
Age	-	-	-	4.4	-0.84–9.65	0.1	5.34	-3.98–14.67	0.26
Education level	5.88	-2.09–13.86	0.14	-4.41	-9.76–0.94	0.1	-	-	-
Race	-5.88	-13.68–2.07	0.14	-	-	-	-	-	-
Socioeconomic status	-	-	-	-	-	-	6.11	-3.07–15.3	0.19
CD4 T-cell	-	-	-	-	-	-	12.33	2.37–22.28	0.01
Viral load	-	-	-	-	-	-	-0.8	-11.34–9.73	0.88
Therapy	-13.81	-25.16–2.46	0.01	-	-	-	10.22	-1.1–21.54	0.07

SAE: Specialized Care Service; D: difference

Significance level: p < 0.05.

## DISCUSSION

In this study, we analyzed the QOL and association between socioeconomic and clinical aspects and healthy living habits in PLWHA. We saw that the most affected domains of QOL were financial concerns, concerns about confidentiality, general function, and satisfaction with life, which were associated, respectively, with the variables: socioeconomic status and physical exercise, therapy, and only physical exercise for the last two domains (general function and satisfaction with life).

The negative results for financial concerns and concerns about confidentiality corroborate with studies that have used the HAT-QoL and identified negative behavior in these domains^5^ ^,^ ^6^ ^,^ ^10^. Regarding the association between socioeconomic status and financial concern, there is enough consistency, as the group had the highest number of persons classified as of low income, agreeing with Passos and Souza^11^ who have assessed 625 PLWHA and found worse QOL for individuals of lower socioeconomic class (p < 0.001). The rationale for such a relationship would be the fact that low-income persons present more difficulties regarding survival (housing, food, and health-related services). These data corroborate with other studies that have identified lower scores in these domains of QOL and low income in the groups^5^ ^,^ ^12^ ^,^ ^13^.

The domain of concern with confidentiality was associated with only the variable of therapy, corresponding to 86.8% of the persons taking antiretroviral drugs. This result is not in agreement with the study of Passos and Souza, who have identified worse QOL associated with “not taking antiretroviral drugs”^11^. However, we can infer from the result, as the literature also states that the use of antiretroviral drugs increase the concerns with confidentiality by fear of revealing the medical condition to family, friends, and co-workers. Despite the information about the disease, prejudice is present and compromises the QOL of PLWHA^14^, making them adopt a “double life”, also affecting the demand for health care^5^ ^,^ ^12^ ^,^ ^13^ ^,^ ^15^ ^-^ ^17^.

General function and satisfaction with life were associated with the variable of physical exercise and were the subsequent domains that presented the lowest averages. The justification for this is due to the negative behavior found by not practicing physical exercise as a healthy habit. There are reports in the literature on the association between QOL of persons who exercise, exactly because of the changes in lifestyle, which allow the improvement of body composition, metabolism efficiency, joint mobility, posture, cognitive functions, perception of self-image, and socialization, thus improving the overall function and satisfaction with life^18^ ^,^ ^19^.

The domains of health concerns, acceptance of HIV, concerns about medication, and sexual function presented positive averages for QOL.

The domain of health concerns was associated with age, physical exercise, and CD4 T-cell. For the first result, we observed that the association presented positive behavior of QOL with advancing age in this study. This result differs from that found by Soares et al.^5^, who have identified that younger PLWHA showed better QOL. The association with physical exercise is also in disagreement with the literature, as the sample consisted of a higher proportion of persons who do not practice physical exercise. A previous study^6^ has shown the reverse, as the persons who were classified as inadequately active showed losses for QOL. However, the association of the domain of health concern with CD4 T-cell can justify the positive behavior of QOL, as this parameter is relevant for the health condition of PLWHA^10^. In our study, most persons showed CD4 T-cell count above 350 cells/mm^3^, which reassures the PLWHA, as this value reduces the concerns about the prognosis of death^10^.

In the analysis of the domain of acceptance of HIV, we observed association with “being over 37 years” and “no practice of physical exercise”. The association with age can arise from the fact that older persons are more mature and thus better accept their health condition^9^. As for exercise, we found disagreement with the literature, as PLWHA classified as physically active presented the best scores for QOL^6^, and the variable “no practice of physical exercise”, in our sample, is not the predominant factor for the positive behavior of the QOL.

The domain of concerns about medication was positive for QOL and was associated with gender (male predominance), age (> 37 years), physical exercise (no practice) and CD4 T-cell (> 350 cells/mm^3^). These data agree with the study of Passos and Souza, who have identified worse QOL associated with female and age (< 47 years)^11^, suggesting that gender, age, and clinical parameters determine the QOL of PLWHA. Regarding the association with physical exercise, another study has observed contrary behavior, in which the domain of concern about medication was less compromised in PLWHA who practiced physical exercise. Thus, we believe that the results found show a non-causal relationship, as the literature indicates that PLWHA who have a healthy lifestyle, as practitioners of physical exercise, are those who care more about their health and have better adherence to antiretroviral drugs, therefore not compromising the QOL in this domain^6^.

Finally, among the analysis of QOL, the domain of sexual function presented one of the best averages. High score or better QOL in this aspect is due to the associations found with males, leading us to two hypotheses: either the men actually have a healthy sex life, or, because of cultural issues or even shame, men deny the impairment of their sexual life^6^. As for the association with the higher values in CD4 T-cell count, the better the immunological parameters, the lower is the risk of HIV transmission. The high CD4 T-cell count also allows the individuals to live better with the disease and rebuild their life projects and affective-sexual bonds^20^ ^,^ ^21^. However, there is controversy regarding the sexual function of PLWHA, as losses are also reported, given that the infection promotes changes that make the subject either avoid their affective relationships^5^.

The main limitation of the study arises from the impossibility of causal inferences, as it is a cross-sectional study. However, the study shows many determinants for the QOL of PLWHA, emphasizing that, when socioeconomic and clinical factors and life habits are not adequate, losses in quality of life can be emphasized.

We conclude that the quality of life of persons living with HIV/AIDS presents losses, mainly in the financial and confidentiality areas, followed by general function of the body and satisfaction with life. Additionally, the domains of general function, satisfaction with life, health concerns, acceptance of HIV, financial concerns, and concerns about medication are associated with no exercise, showing that this factor, as well as some socioeconomic factors, are determinants for the QOL of PLWHA.

From the premise that both the practice of physical exercise and socioeconomic factors are determinant for the quality of life of the population studied, we suggest the creation of public policies focused on both aspects, such as the encouragement of physical exercise and good nutrition. These guidelines would support the traditional treatment, providing the complete follow-up of PLWHA, from the strengthening of the multidisciplinary team. Such assistance would address the clinical, psychological, physical, and social aspects, which directly reflect on the QOL of the persons living with HIV/AIDS.
